# ICCTax: a hierarchical taxonomic classifier for metagenomic sequences on a large language model

**DOI:** 10.1093/bioadv/vbaf257

**Published:** 2025-10-15

**Authors:** Yichun Gao, Jiaxing Bai, Feng Zhou, Yushuang He, Ying Wang, Xiaobing Huang

**Affiliations:** Department of Automation, National Institute for Data Science in Health and Medicine, State Key Laboratory of Mariculture Breeding, Xiamen Key Laboratory of Big Data Intelligent Analysis and Decision, Xiamen University, Fujian 361102, China; Department of Automation, National Institute for Data Science in Health and Medicine, State Key Laboratory of Mariculture Breeding, Xiamen Key Laboratory of Big Data Intelligent Analysis and Decision, Xiamen University, Fujian 361102, China; Department of Automation, National Institute for Data Science in Health and Medicine, State Key Laboratory of Mariculture Breeding, Xiamen Key Laboratory of Big Data Intelligent Analysis and Decision, Xiamen University, Fujian 361102, China; Department of Automation, National Institute for Data Science in Health and Medicine, State Key Laboratory of Mariculture Breeding, Xiamen Key Laboratory of Big Data Intelligent Analysis and Decision, Xiamen University, Fujian 361102, China; Department of Automation, National Institute for Data Science in Health and Medicine, State Key Laboratory of Mariculture Breeding, Xiamen Key Laboratory of Big Data Intelligent Analysis and Decision, Xiamen University, Fujian 361102, China; Department of Medical Oncology, Fuzhou First Hospital Affiliated with Fujian Medical University, Fuzhou, Fujian 350005, China

## Abstract

**Motivation:**

Metagenomic data increasingly reflect the coexistence of species from Archaea, Bacteria, Eukaryotes, and Viruses in complex environments. Taxonomic classification across the four superkingdoms is essential for understanding microbial communities, exploring genomic evolutionary relationships, and identifying novel species. This task is inherently imbalanced, uneven, and hierarchical. Genomic sequences provide crucial information for taxonomy classification, but many existing methods relying on sequence similarity to reference genomes often leave sequences misclassified due to incomplete or absent reference databases. Large language models offer a novel approach to extract intrinsic characteristics from sequences.

**Results:**

We present ICCTax, a classifier integrating the large language model HyenaDNA with complementary-view-based hierarchical metric learning and hierarchical-level compactness loss to identify taxonomic genomic sequences. ICCTax accurately classifies sequences to 155 genera and 43 phyla across the four superkingdoms, including unseen taxa. Across three datasets built with different strategies, ICCTax outperforms baseline methods, particularly on Out-of-Distribution data. On Simulated Marine Metagenomic Communities datasets from three oceanic sites, DairyDB-16S rRNA, Tara Oceans, and wastewater metagenomic datasets, it demonstrates strong performance, showcasing real-world applicability. ICCTax can further support identification of novel species and functional genes across diverse environments, enhancing understanding of microbial ecology.

**Availability and implementation:**

Code is available at https://github.com/Ying-Lab/ICCTax.

## 1 Introduction

Microbial communities exhibit high complexity in their composition and dynamics. Their taxonomy classification is crucial to uncover ecological structure. Metagenomic sequencing data often provides essential information to decipher the composition of the community. In marine ecosystems, metagenomic sequencing data capture the genomic sequences from bacteria, archaea, viruses, and microeukaryotes like algae, fungi, and protozoa. In human-associated microbial community, metagenomic data include bacteria, viruses, a small presence of archaea, occasional eukaryotes of fungi, and frequent admixture with host genomic sequences. Therefore, it is essential to identify the taxonomy metagenomic sequences in the four superkingdoms of Archaea, Bacteria, Eukaryotes, and Viruses, to decipher community composition and structure, discover a large proportion of unknown species, and helping to understand the functionality of the microbial community.

Genomic sequences provide essential data for taxonomic classification, yet several challenges remain. First, genomic similarities among organisms, such as shared gene structures between archaea and bacteria, and viral integration of host sequences (Roux *et al.* 2015), lead to contigs sharing homologous sequence regions, which complicates taxonomic classification. Second, microorganisms exhibit high mutation rates due to genetic diversity and environmental factors. The mutation rate in bacteria is generally around $5\times 10^{-9}$ substitutions per base pair per cell division (Turrientes *et al.* 2013), and DNA viruses exhibiting rates ranging from $10^{-8}$ to$10^{-6}$ substitutions per nucleotide site per cell infection (s/n/c), and RNA viruses showing rates between $10^{-6}$ and $10^{-4}$ s/n/c (Sanjuán *et al.* 2010). High mutations lead to mismatches in the alignment for taxonomic identification. Lastly, incomplete reference databases hinder accurate classification, as 86% of eukaryotic species and 91% of ocean species remain undescribed, and current bacterial and archaeal databases fail to capture full species diversity (Mora *et al.* 2011, Louca *et al.* 2019).

Reference-based methods and learning-based methods have been proposed for these challenges. Reference-based methods, including Kraken (Wood and Salzberg 2014, Wood *et al.* 2019), MMseqs2 (Steinegger and Söding 2017), sourmash (Brown and Irber 2016), and Minimap2 (Li 2018), MetaPhlAn4 (Blanco-Míguez *et al.* 2023) and Contig Annotation Tool (CAT) (Von Meijenfeldt *et al.* 2019) classify accurately through efficient searching algorithms, but reference database reliance limits their ability to classify underrepresented or missing sequences. In contrast, learning-based methods, such as DeepMicrobes (Liang *et al.* 2020), offer a more flexible approach by learning features from training sequences, reducing reliance on reference databases. DeepMicrobes uses *k*-mer embeddings (*k *= 12) as features and processes them using bidirectional LSTMs. However, these methods struggle to capture global sequence features, limiting their performance when processing longer or highly variable sequences.

Large language models, which can treat genomic sequences as natural language, offer a more powerful solution to integrate and extract complex genomic information. Pre-trained models using Transformer architectures (Vaswani 2017) are emerging. For instance, BERTax (Mock *et al.* 2022) adopts the Bidirectional Encoder Representations from Transformers (BERT) (Devlin *et al.* 2019) and tokenizes DNA sequences into 3-mers, facilitating efficient classification across multiple taxonomic levels. Hyena architecture (Poli *et al.* 2023) introduces implicit long convolutions, which significantly reduce the time and space complexity challenges that traditional Transformer models face. HyenaDNA (Nguyen *et al.* 2024), built on the Hyena architecture, treats single nucleotides as tokens, achieving unprecedented single-nucleotide resolution and thereby better capturing subtle genetic variations.

However, from an information view, taxonomy classification is essentially a highly imbalanced, uneven, and hierarchical classification problem. Taxonomy, including Superkingdom, Phylum, Class, Order, Family, Genus, and Species, forms a hierarchical tree, where each level represents a distinct taxonomic clade, and intra-class compactness reflects the similarity between instances within the same clade, with higher compactness resulting in clearer class boundaries. However, the high imbalanced species numbers across hierarchical taxonomic clades and levels lead to poor performance in few-shot clades. Furthermore, when faced with Out-of-Distribution (OOD) data, where the model has never seen the species from a genus or a novel genus, the model’s inability to recognize unseen features leads to a decline in classification accuracy. Therefore, effectively addressing imbalanced data and OOD challenges in hierarchical classification, and enhancing intra-class compactness, is crucial for improving model generalization and classification accuracy.

To address these challenges, we present ICCTax, a hierarchical taxonomic classifier that uses HyenaDNA to extract comprehensive feature information from genomic sequences and classify across four superkingdoms, encompassing 43 phyla and 155 genera. Since original sequences and their reverse-complementary sequence belong to the same clade, ICCTax utilizes a complementary-view-based hierarchical metric learning architecture to capture their intrinsic equivalence and ensure similar predicted distributions in the feature space, while incorporating a hierarchical-level compactness loss to fine-tune HyenaDNA, enhancing classification performance by improving intra-class compactness across multiple taxonomic levels. We conducted a comprehensive evaluation of ICCTax on In-Distribution (ID), OOD, and Complete datasets. The ID and OOD datasets contain the same genomes but differ in partitioning. In the ID dataset, the testing genomic sequences are from genera included in the training set, while in the OOD dataset, testing sequences are from genera not seen during training. The different construction strategies for ID and OOD datasets aim to simulate distinct application scenarios: ID involves query sequences from known genera, and OOD involves sequences from novel species and genera. Finally, ICCTax is trained and tested on a Complete dataset, including 11 unknown phyla. ICCTax exhibits superior performance over the baseline methods. On the Complete dataset, ICCTax achieves strong performance across 43 phyla and 155 genera, showcasing its ability to handle fine-grained classification tasks. On the Simulated Marine Metagenomic Communities datasets, constructed from three ecologically distinct oceanic sites with varying taxonomic compositions and sequencing error rates, ICCTax consistently outperforms baseline methods, demonstrating its robustness. Additionally, ICCTax experiments on the 16S rRNA sequencing dataset highlight its effectiveness in classifying prokaryotic, and its predictions on the Tara Oceans and wastewater metagenomic datasets show its capability in handling real-world environmental data. The main contributions of this study are as follows:

1. ICCTax uses HyenaDNA to extract comprehensive feature information and uses its decoder to build an all-in-one architecture (Rojas-Carulla *et al.* 2019) that connects higher-level prediction embeddings with DNA sequence embeddings to achieve lower-level predictions. This design enables generalize to unseen or unknown species, mitigating performance degradation and addressing the OOD challenge.
2. ICCTax introduces a hierarchical-level compactness loss to address the difficulties in distinguishing closely related taxa caused by high sequence similarity across different taxonomic clades. For each class, a class center is initialized, and the distance between the embedding of each sequence and its corresponding class center is penalized. The class centers are then iteratively updated to minimize the distance, improving classification accuracy.
3. ICCTax employs Kullback-Leibler Divergence (KLD) (Kullback 1997) as a distance metric to establish the complementary-view-based hierarchical metric learning architecture. By capturing the inherent feature similarity between sequences and their reverse complements and quantifying their differences, it addresses the challenges posed by the fuzzy boundaries resulting from genetic diversity within species.

## 2 Methods

**Figure 1.**
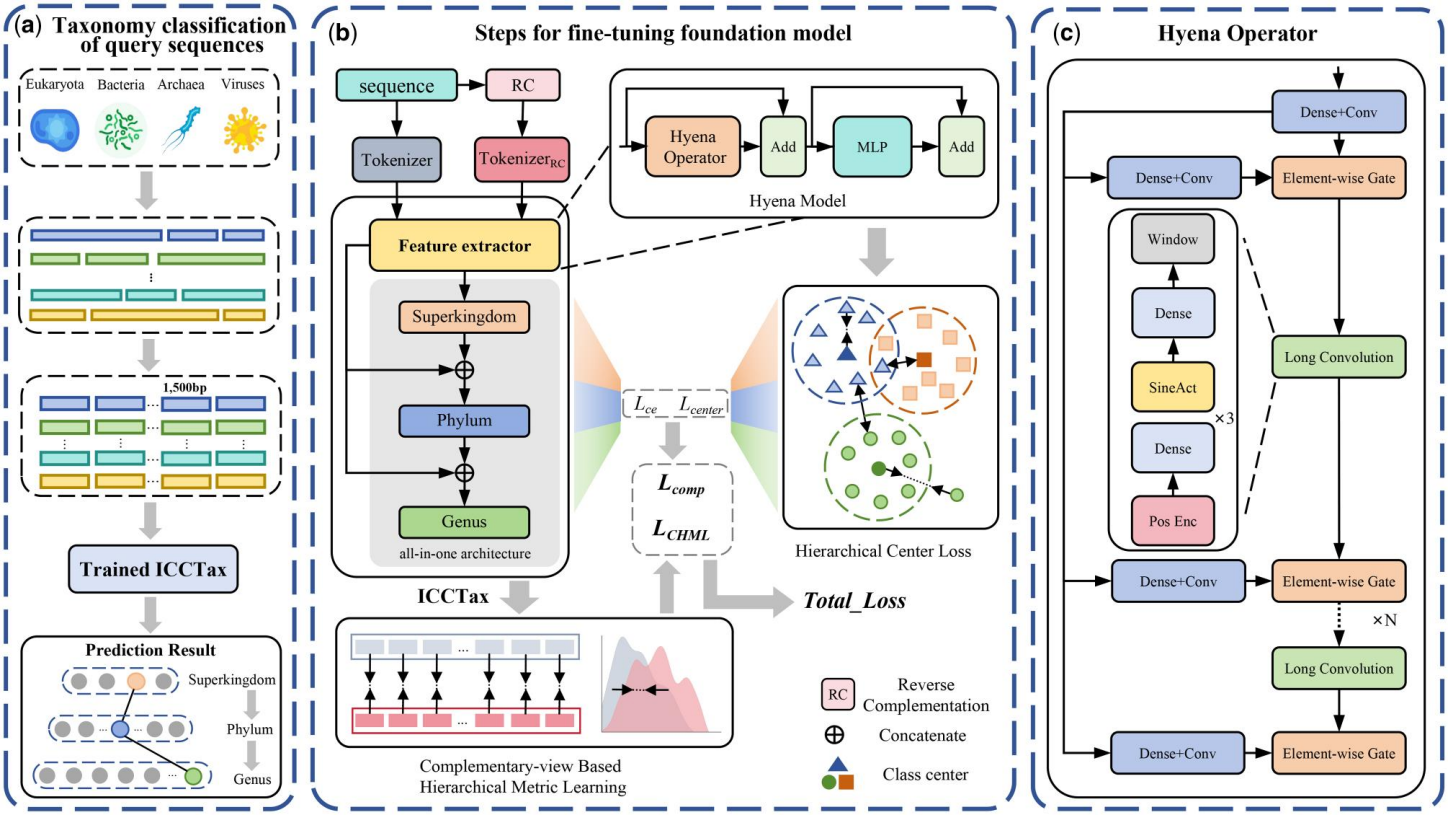
Overview of the proposed ICCTax for taxonomy classification. (a) Taxonomic classification of query sequences. (b) Fine-tuning pipeline of ICCTax, which integrates hierarchical metric learning with compactness loss and employs the HyenaDNA decoder for multi-level classification. (c) HyenaDNA Operator Architecture.

In this study, we propose ICCTax, which performs hierarchical taxonomy classification on 1500 bp contigs trimmed from genomic fragments obtained through metagenomic sequencing, addressing imbalanced data, and OOD challenges. Figure 1 shows the architecture of ICCTax. ICCTax employs HyenaDNA as its foundational model to extract feature representations from long genomic sequences, with its operator architecture shown in Fig. 1c. Figure 1b illustrates the fine-tuning process of HyenaDNA, where ICCTax uses KLD to build a complementary-view-based hierarchical metric learning architecture that captures intrinsic equivalence and incorporates a hierarchical-level compactness loss to enhance model performance. Additionally, ICCTax uses HyenaDNA's decoder to build an all-in-one architecture that allows output layers to access the model’s internal features and predictions from higher taxonomic levels, while treating the training process at each classification level as an independent task and incorporating learnable parameters to adjust task weights, optimizing multi-task learning performance.

### 2.1 HyenaDNA

HyenaDNA is a large language model specifically designed for genomic sequence analysis, with the Hyena operator functioning as its central computational module (Nguyen *et al.* 2024), as shown in Fig. 1c. Based on HyenaDNA, we build an all-in-one architecture using its decoder. This architecture enables information flow across different hierarchical levels by connecting higher-level prediction embeddings with DNA sequence embeddings to facilitate lower-level predictions, effectively addressing the OOD challenge.

Formally, the tokenized sequences $x\in \;R^{L}$ (where L denotes the length) is mapped into three linear projections, which are processed using short convolutions (Poli *et al.* 2023) as follows:


(1) $$\{q,k,v\}=\mathrm{DepthwiseConv}1d(W_{i}x,s_{i}),$$

where $W_{i}$ refers to the linear projection matrices $W_{q}$, $W_{k}$, and $W_{v}$, mapping the input x into query, key, and value spaces, respectively. $s_{i}$ represents the corresponding short convolution filter applied in the depthwise convolution, and DepthwiseConv1d(·) refers to the one-dimensional depthwise convolution operation applied to each channel.

After obtaining q, k, and v, the Hyena operator H processes these projections using implicit long convolutions and element-wise gating, as described by the following equations:


(2) (q,k,v)⟼H(q,k)v,

(3) $$H(q,k)=D_{k}T_{h}D_{q},$$

where $T_{h}\in R^{L\times L}$ is the Toeplitz kernel matrix (Nguyen *et al.* 2024) generated by a learnable implicit long convolution, produced as the output of a neural network. The kernel matrix $T_{h}$ is parameterized by:


(4) $${\left(T_{h}\right)}_{ij}=h_{i-j}.$$

The value $h_{p}$ is generated by a neural network γθ, which uses position index p and optional positional encoding as inputs:


(5) $$h_{p}=\gamma \theta (p).$$

This feature enables the operator H to manage extensive sequences efficiently without growing linearly in parameter count (Nguyen *et al.* 2024). Additionally, the matrices $D_{q}$, $D_{k}\;\in \;R^{L\times L}$ are constructed by placing q and k along the diagonals, with definitions as follows:


(6) $$D_{q}=\mathrm{Diag}(q),$$

(7) $$D_{k}=\mathrm{Diag}(k),$$

where the function Diag(·) converts the vector into a diagonal matrix for element-wise gating in the model.

After obtaining the embedding representation of the DNA sequence, the decoder generates higher-level prediction outputs, which are expanded to match the dimensions of the next hierarchical level and concatenated with the DNA sequence embeddings. This iterative process continues until the final classification level is reached, ensuring that each level benefits from both sequence features and hierarchical context, forming the all-in-one architecture. The procedure is outlined as follows:


(8) $$O_{n}=\{\begin{array}{c} {\mathrm{Dec}}_{n}(\mathrm{Emb}),\;if\;n=1\\ {\mathrm{Dec}}_{n}(\mathrm{concat}(Emb,O_{n-1}\;⨂\;1_{L+2})),\;\mathrm{if}\;n>1 \end{array},$$

where Emb represents the embedding of the sequence. At each hierarchical level, the output $O_{n}$ is generated by the nth decoder ${\mathrm{Dec}}_{n}$. This output is multiplied with the sequence embeddings using the tensor product “⨂” to align the dimensions for expansion. The expanded embeddings are then concatenated with the sequence embeddings at the next level to form new inputs.

### 2.2 Complementary-view based hierarchical metric learning

Taxonomic classification faces a significant challenge due to the genetic diversity across different clades, leading to lower intra-class compactness. Even sequences within the same taxonomic group can show significant feature differences due to variations in composition, structure, or sequencing errors, causing semantic ambiguity where sequences may not be correctly matched by the classification model.

To address this issue, we propose a complementary-view based hierarchical metric learning architecture. From the perspective of reverse-complement sequences, this architecture ensures the original sequence and its reverse-complement, which belong to the same clade at the same hierarchical level, have similar predicted distributions in the feature space. The key innovation is using a metric-based approach to learn a similarity function that captures the inherent equivalence between these sequence pairs. The architecture uses KLD as a distance metric, which is advantageous for measuring relative information due to its asymmetry. This asymmetry helps capture the subtle differences in the predicted distributions of the original and reverse-complement sequences, thus ensuring a better alignment in the feature space. Thus, KLD is an ideal choice for our architecture, defined as:


(9) $$D_{\mathrm{KL}}(P||Q)=\sum_{x}P(x)\log(\frac{P(x)}{Q(x)}),$$

where P(x) represents the predicted distribution of the original sequence and Q(x) the predicted distribution of its reverse-complement sequence. In this study, KLD is used to measure how well their predicted distributions align. Specifically, let $P_{\mathrm{orig}}^{\tau }$ and $P_{\mathrm{comp}}^{\tau }$ be the predicted probability distributions of the original sequence and the corresponding reverse complementary sequence, where each probability represents the likelihood of the sequence belonging to a specific class at the taxonomic level τ∈{superkingdom,phylum,genus}.

The KLD between $P_{\mathrm{orig}}^{\tau }$ and $P_{\mathrm{comp}}^{\tau }$ serves as the distance metric in a shared feature space. Since the original and reverse complement sequences share identical taxonomic labels at each level, the model is expected to predict identical distributions for both, despite potential differences in their feature representations. The optimization objective is to minimize the KLD across the selected taxonomic levels. This is achieved by calculating the divergence between $P_{\mathrm{orig}}^{\tau }$ and $P_{\mathrm{comp}}^{\tau }$, quantifying how well the model’s predictions for both sequences are aligned. The corresponding loss function at the τ -level can be expressed as:


(10) $$L_{\mathrm{CHML}\_\tau }=D_{\mathrm{KL}}(P_{\mathrm{orig}}^{\tau }||P_{\mathrm{comp}}^{\tau }).$$

Optimizing ICCTax with $L_{\mathrm{CHML}}$ enhances its ability to capture the biological equivalence between original and complementary sequences, while retaining taxon-specific discriminative features.

### 2.3 Compactness loss

In taxonomic classification, imbalanced data and high mutation rates cause intra-class variations across different taxonomic levels, making it difficult for traditional models relying on cross-entropy loss to distinguish closely related classes. To improve classification accuracy and promote feature compactness, we designed a compactness loss by combining cross-entropy loss with center loss.

The cross-entropy loss measures the difference between the predicted probability distribution and the true label, encouraging ICCTax to improve classification accuracy by bringing the predicted probabilities closer to the true labels (Zhang and Sabuncu 2018). For three taxonomic level, the formula for cross-entropy loss is as follows:


(11) $$L_{\mathrm{ce}\_\tau }=-{\sum_{i=1}^{N}\log(p_{i})y}_{i},$$

where $y_{i}$ is the true label and $p_{i}$ is the predicted probability for class i at level τ.

In addition, center loss (Wen *et al.* 2016) enhances ICCTax’s discriminative ability by learning a center for the deep features of each class and penalizing the distances between these deep features and their corresponding class centers. This method is particularly effective for taxonomy classification tasks, as it improves intra-class compactness, which is essential for distinguishing between closely related taxonomic clades. The center loss at the taxonomic level τ is formulated as:


(12) $$L_{\mathrm{center}\_\tau }=\frac{1}{2}\sum_{i=1}^{m}||x_{i}-c_{yi}|{|}_{2}^{2},$$

where the $c_{yi}\in R^{d}$ represents the class center of $y_{i}\;$deep features. The updates are performed based on mini-batches, where the center points are updated by averaging the features of the corresponding class, though some may remain unchanged in certain cases. Additionally, to mitigate large perturbations caused by a small number of mislabeled samples, a scalar α is introduced to control the learning rate for the centers. The gradients of the center loss $L_{\mathrm{center}}$ with respect to $x_{i}$ and update the equation of $c_{yi}$ are computed as:


(13) $$\frac{\partial L_{\mathrm{center}\_\tau }}{\partial x_{i}}=\frac{1}{m}(x_{i}-c_{yi}),$$

(14) $$\Delta c_{j}=\frac{\sum_{i=1}^{m}\delta (y_{i}=j)\cdot (c_{j}-x_{i})}{1+\sum_{i=1}^{m}\delta (y_{i}=j)},$$

(15) δ(yi=j)={1, yi=j0, otherwise,

Here $\delta (y_{i}=j)$ is an indicator function that selects the samples belonging to class j, ensuring that only relevant features contribute to the update of the class center $c_{j}$. The $\Delta c_{j}$ captures the aggregated deviation between the current center and the deep features of class j, normalized by the number of contributing samples. The update rule for the class center is defined as:


(16) $$c_{j}^{\left(t+1\right)}=c_{j}^{\left(t\right)}-\eta \;\Delta c_{j},$$

where $c_{j}^{\left(t\right)}$ denotes the center for class j at training iteration t, and η is the learning rate controlling the update step size. Thus, compactness loss is composed of center loss and cross-entropy loss, applied at each taxonomic level. The total loss function can be expressed as:


(17) $$L_{\mathrm{comp}\_\tau }=L_{\mathrm{ce}\_\tau }+\lambda_{\tau }L_{\mathrm{center}\_\tau },$$

where the coefficient $\lambda_{\tau }$ is used to adjust the relative importance of the two components in the proposed training objective at each level. Compactness loss enhances the model’s performance, effectively addressing the challenge of intra-class variations.

### 2.4 Total loss function

The parameters of ICCTax are optimized by combining compactness loss $L_{\mathrm{comp}}\;$and complementary-view-based hierarchical metric learning loss $L_{\mathrm{CHML}}$ across multiple taxonomic levels. Specifically, ICCTax computes separate losses for each of the three taxonomic levels: superkingdom, phylum, and genus. Let T={superkingdom,phylum,genus} denote the set of all considered taxonomic levels, and let τ∈T represent a specific taxonomic level in the hierarchical classification. For each level τ, we introduce a learnable weight $\zeta_{\tau }$ to adaptively balance the corresponding losses, along with a logarithmic regularization term that prevents $\zeta_{\tau }$ from collapsing toward zero. The total loss is then defined as the summation over all levels:


(18) $$\mathrm{Loss}=\sum_{\tau \in T}\frac{1}{2}\zeta_{\tau }^{2}({L_{\mathrm{comp}\_\tau }+\alpha }_{\tau }L_{\mathrm{CHML}\_\tau })+\ln\left(1+\zeta_{\tau }^{2}\right),$$

where $\alpha_{\tau }$ is a hyperparameter that controls the trade-off between $L_{\mathrm{comp}\_\tau }$ and $L_{\mathrm{CHML}\_\tau }$, and $\zeta_{\tau }^{2}\;$updated through gradient descent to minimize the overall objective. This hierarchical and comprehensive loss function enforces intra-class compactness at each taxonomic level, improving generalization and classification accuracy across varying data.

## 3 Experiments

### 3.1 Datasets

**Figure 2.**
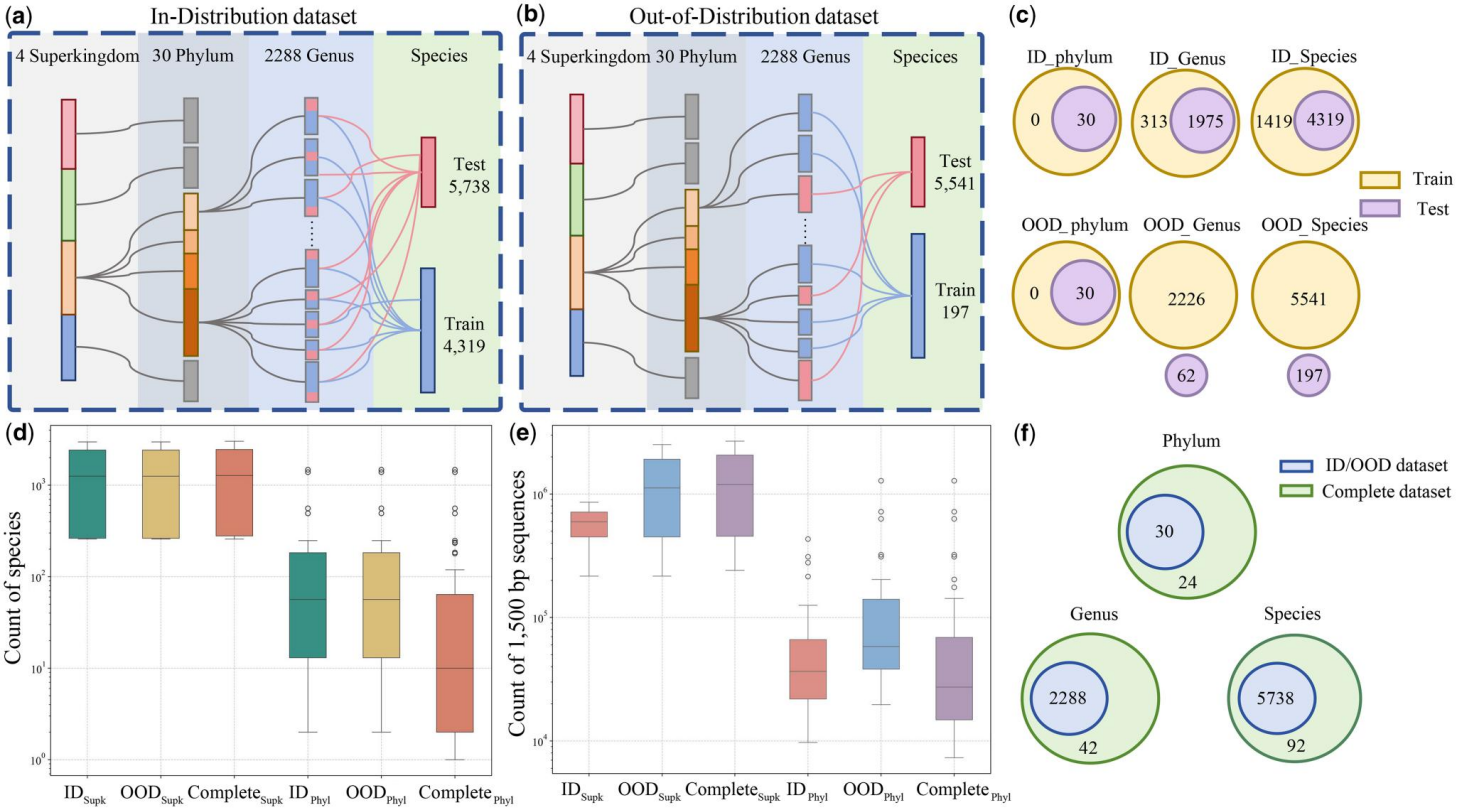
Selection process for ID and OOD datasets, along with dataset statistics. (a) ID dataset: 2000 sequences per phylum for testing, remainder for training. (b) OOD dataset: Sequences from each genus are fully assigned to either the training or test set. (c) Differences in genera and species between the ID and OOD training/testing sets, containing the same genomes but partitioned differently. (d, e) Species and sequence distributions across ID, OOD, and Complete datasets. (f) Clade count differences between Complete and ID/OOD datasets before processing.

This study utilizes multiple datasets of varying levels and scenarios, which are constructed by the study of BERTax (Mock *et al.* 2022). These datasets include an ID dataset, an OOD dataset, and a Complete dataset, each consisting of 1500 bp genomic sequences extracted from the genomes of Archaea, Bacteria, Eukaryotes, and Viruses. The ID and OOD datasets are constructed with distinct strategies to represent two application scenarios, with the partitioning differences shown in Fig. 2c. Figure 2f illustrates the clade count differences at various classification levels between the Complete dataset before processing and the ID/OOD datasets, with the latter being subsets of the former. The training configurations for the ID/OOD and Complete datasets are detailed in Supplementary Section 1. Among them, the ID and OOD datasets are specifically designed experimental data, while ICCTax trained on the Complete dataset will be directly used as a classifier for various future scenarios, including the Simulated Marine Metagenomic Communities, DairyDB-16S rRNA, Tara Oceans and wastewater metagenomic datasets, thereby enabling a comprehensive evaluation across both controlled and real-world metagenomic conditions. Detailed descriptions, construction procedures, and statistics of all seven datasets are provided in Supplementary Section 2.

### 3.2 Experiments on ICCTax performance

The eight state-of-the-art methods are compared, including six reference-based methods: MMseqs2, Minimap2, Kraken2, sourmash, MetaPhlAn4, and CAT, and two learning-based methods: DeepMicrobes and BERTax. To assess the effectiveness of ICCTax, we conducted a comprehensive comparison with these methods using three different test datasets. Runtime performance is reported in Supplementary Section 3.

#### 3.2.1 Performance on the ID dataset and OOD dataset

This section evaluates the classification performance of all methods on the ID and OOD datasets at both the superkingdom and phylum levels. The performance is evaluated using Accuracy (Acc) and macro average precision (AveP) (Supplementary Section 4). Compared to Acc, AveP provides a more balanced perspective by considering the contribution of each class individually. As shown in Table 1 and in the micro- and macro-averaged AUC results (Tables S3 and S4), ICCTax outperforms all other methods across both datasets. Compared to MMseqs2, ICCTax improves Acc by 11.18% and 5.34%, and AveP by 7.58% and 11.31% at the superkingdom and phylum levels, respectively. In comparison to DeepMicrobes, ICCTax achieves a 2.71% higher Acc and a 10.35% higher AveP at the phylum level. MetaPhlAn4 showed suboptimal performance, likely due to its reliance on a fixed marker gene database and the lack of support for custom user-defined references, which limits its ability to detect unseen taxa.

**Table 1.** Comparison of the Acc and macro AveP on the ID and OOD datasets.^a^

	ID dataset				OOD dataset			
	Superkingdom		Phylum		Superkingdom		Phylum	
	Acc	AveP	Acc	AveP	Acc	AveP	Acc	AveP
MMseqs2	87.13	92.19	85.09	85.66	47.89	62.76	39.61	41.36
Minimap2	75.55	86.12	75.25	76.06	18.77	44.12	15.33	20.03
Kraken2	75.97	86.26	75.05	75.99	19.56	44.36	14.39	19.50
sourmash	6.91	30.69	6.89	9.04	0.16	25.14	0.15	3.48
DeepMicrobes	96.68	97.18	87.72	86.62	68.39	67.25	41.95	36.61
BERTax	94.78	95.65	85.55	83.88	88.95	90.06	60.10	54.10
MetaPhlan4*	2.16	NA	0.94	NA	1.90	NA	1.02	NA
CAT	82.23	NA	77.22	NA	65.89	NA	53.22	NA
ICCTax	**98.31**	**99.77**	**90.43**	**96.97**	**92.04**	**97.61**	**61.53**	**70.17**

^a^Since MetaPhlan4 and CAT do not provide probability scores for classification, AveP is unapplicable (NA) to these methods. MetaPhlAn4 was evaluated using its official pre-built reference database, as indicated by (*).

The bold values in the table indicate the best performance.

All methods experienced performance degradation on the OOD dataset. However, alignment-based methods decline significantly when handling unseen sequences due to the limitations of their alignment mechanisms with unfamiliar data. Among learning-based methods, while DeepMicrobes performs strongly on the ID dataset, BERTax outperforms it on the OOD dataset, showcasing superior robustness in handling unseen sequences. ICCTax shows greater robustness, with Acc declining by only 6.48% and AveP declining by just 2.16% from the ID dataset to the OOD dataset at the superkingdom level. Moreover, ICCTax outperforms the other methods at the phylum level. This stability proves that ICCTax is a reliable choice for classification tasks under varying conditions. Moreover, 95% bootstrap confidence intervals (CIs) for Acc and AveP on both the ID and OOD datasets (Figs. S3 and S4) further confirm the robustness of these results.

#### 3.2.2 Performance on the complete dataset

The performance of ICCTax is also compared with other methods on the Complete dataset. The Complete dataset incorporates a greater variety and quantity of closely related sequences from the reference database, while also introducing increased data distribution imbalance.

Table 2 demonstrate that ICCTax achieves the highest AveP at the superkingdom and phylum levels. At the genus level, ICCTax attains the highest AveP among all learning-based methods (67.20%), outperforming BERTax (66.92%) and DeepMicrobes (66.43%), thereby demonstrating its effectiveness in fine-grained taxonomic classification. The superior performance of ICCTax stems from its ability to effectively learn the feature centers for various samples, which enhances intra-class compactness. Additionally, the interaction of information across hierarchical levels strengthens its generalization capability across diverse data.

**Table 2.** Comparison of the macro AveP on the Complete dataset.

AveP	Superkingdom	Phylum	Genus
MMseqs2	96.94	92.90	**74.76**
Minimap2	93.46	86.71	66.68
Kraken2	93.65	87.13	70.56
sourmash	31.04	8.00	3.07
DeepMicrobes	98.13	92.11	66.43
BERTax	98.62	95.10	66.92
ICCTax	**99.76**	**96.95**	67.20

The bold values in the table indicate the best performance.

#### 3.2.3 Evaluation of ICCTax’s classification performance across taxonomic levels

**Figure 3.**
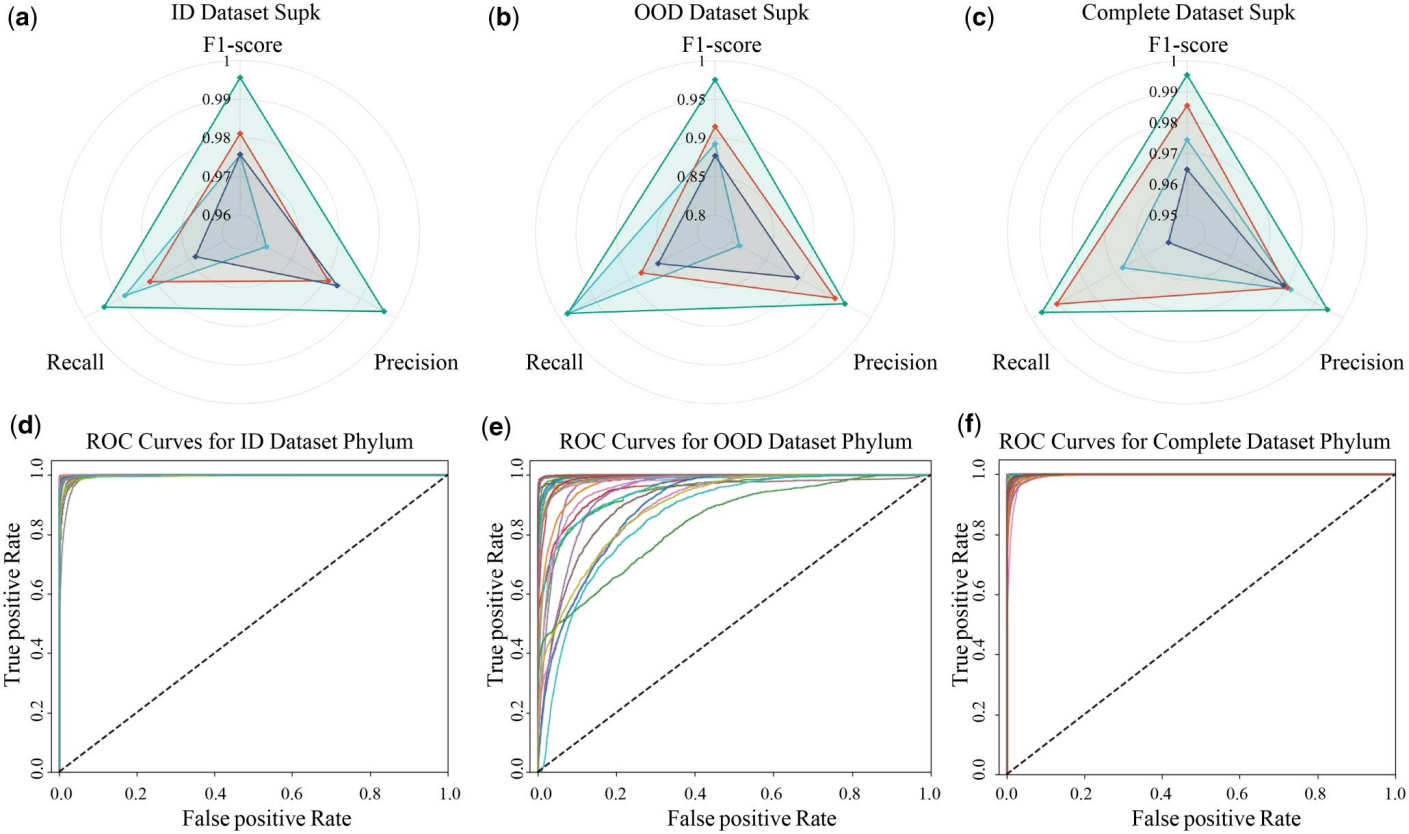
ICCTax performance on three datasets: (a–c) superkingdom-level metrics and (d–f) phylum-level ROC curves.

This section evaluates ICCTax’s classification performance, offering insights into its effectiveness. Figure 3a–c and Fig. S1 highlight ICCTax’s strong performance at the superkingdom level across three datasets. Notably, eukaryotic achieved the highest score, indicating ICCTax’s robust classification ability for this superkingdom. In contrast, viruses, due to their high mutation rates and genomic diversity, exhibit greater variability, resulting in a broader distribution in feature space and making accurate classification more challenging. Further analysis of Fig. 3d and f shows ICCTax’s exceptional performance on the ID and Complete datasets, demonstrating its ability to effectively learn and capture the distribution patterns of each phylum within the feature space.

As shown in Fig. 3e, ICCTax demonstrates strong performance on the OOD dataset, only 8 out of 30 phyla have an AUROC value below 0.95, with the lowest AUROC being 0.8351. Additionally, Fig. S2 shows ICCTax’s performance for each phylum across the three datasets. These results show ICCTax’s robustness in handling complex real-world data while maintaining high accuracy across a broad range of taxonomic groups.

### 3.3 Evaluation on simulated marine metagenomic communities datasets

To better reflect the complexity of real-world clinical and environmental metagenomes, we simulated marine communities with varying phylum-level abundances and introduced different levels of sequencing error. We compared ICCTax with four baseline methods—Minimap2, Kraken2, MMseqs2, and BERTax—on the Simulated Marine Metagenomic Communities dataset. As shown in Fig. S5, ICCTax consistently outperformed other methods across sampling sites (P1, NP3, NP5) and error rates (0%, 0.5%, and 5%), at both the superkingdom and phylum levels. Notably, ICCTax exhibited strong robustness to sequencing noise, maintaining stable classification accuracy even under high error rates. For instance, at site P1 under 5% error rate, ICCTax achieved 99.10% Acc at the superkingdom level and 92.76% at the phylum level, which are 1.68% and 3.51% higher than those of BERTax, respectively. These results highlight ICCTax’s ability to extract discriminative features from noisy reads and generalize effectively across diverse marine community compositions. Additionally, Fig. S5 presents the phylum-level read abundance distributions under the 0% error rate. ICCTax produced abundance profiles that most closely matched the ground truth across all three sites, further demonstrating its effectiveness in reconstructing taxonomic compositions in complex microbial environments.

Furthermore, we evaluated the effect of read length (500–2500 bp) on the simulated P1 with 5% error, and confirmed that ICCTax effectively exploits extended sequence context while maintaining strong performance on short reads (Fig. S6).

### 3.4 Model analysis of ICCTax

#### 3.4.1 Visualization of model embeddings

**Figure 4.**
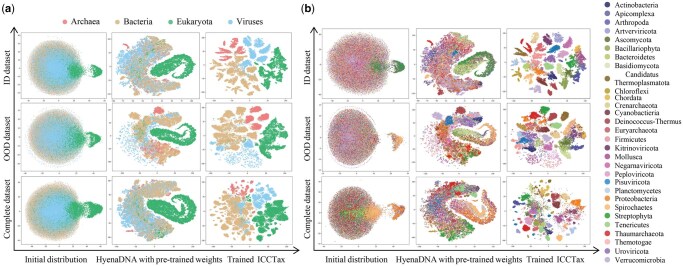
t-SNE visualizations of DNA sequence embeddings generated by ICCTax across the ID, OOD, and Complete datasets. (a) Embeddings at the superkingdom level, and (b) at the phylum level. In the Complete dataset, only the same 30 phyla as those in the ID and OOD datasets are displayed.

To verify the improvement in the ICCTax’s ability to distinguish between different superkingdoms and phyla through its training process, we visualize the embeddings of DNA sequences at different stages using the t-distributed Stochastic Neighbor Embedding (t-SNE) (van der Maaten and Hinton 2008). Figure 4 illustrates ICCTax’s progression in mapping and distinguishing features across different taxonomic levels. Initially, the embeddings of raw sequences display minimal separation. As pre-training advances, discernible distinctions start to appear. Finally, after fine-tuning, the embeddings exhibit clear separation at both the superkingdom and phylum levels. Details on ICCTax interpretability are presented in Supplementary Section 10.

#### 3.4.2 Ablation study

To demonstrate the effectiveness of compactness loss and complementary view-based hierarchical metric learning architecture in enhancing intra-class compactness, we visualize the t-SNE of embedding distributions for the trained HyenaDNA and ICCTax at the phylum level on the ID dataset, as shown in Fig. S7a and b, respectively. These visualizations clearly illustrate that the methods create distinct boundaries between classes while making sequences within the same class more compact. Additionally, Fig. S7c presents box plots of intra-class distance distribution computed within the same embedding space for both models, showing that ICCTax achieves greater intra-class compactness, significantly reducing the intra-class distances compared to HyenaDNA.

So as to assess the factors contributing to ICCTax’s significant improvement, we compared it against the trained HyenaDNA, ICCTax without compactness loss optimization, and ICCTax without complementary view-based hierarchical metric learning, as shown in Fig. S7d. The compactness loss enables ICCTax to focus more on shared features within the same class, while complementary-view-based hierarchical metric learning allows ICCTax to capture more comprehensive and effective information. We further validated the choice of KLD in the ablation study, and the results are reported in Supplementary Section 8.

### 3.5 Performance on 16S rRNA sequences

The 16S rRNA gene is a widely used genetic marker for identifying and classifying bacteria and archaea. Found in the ribosomes of all prokaryotes, it contains both conserved and variable regions (Yarza *et al.* 2014). The conserved regions all ow for broad taxonomic identification, while the variable regions enable species-specific differentiation. The identification and classification of the 16S rRNA gene contribute to the study of microbial diversity, ecology, and taxonomy, and are crucial for advancing our understanding of microbial communities in both environmental and clinical contexts.

Figure S8 demonstrates that ICCTax demonstrates excellent performance on the sequences from the DairyDB-16S dataset. In the seen phylum, ICCTax achieves an Acc of 99.92% at the superkingdom level and 72.44% at the phylum level. In contrast, for the unseen phylum, which represents the OOD data, ICCTax classifies them at a higher level (superkingdom), achieving an accuracy of 99.60%. These results highlight the robustness of ICCTax and its ability to effectively handle both familiar and unseen sequences.

### 3.6 Taxonomic profiling on real metagenomic data

**Figure 5.**
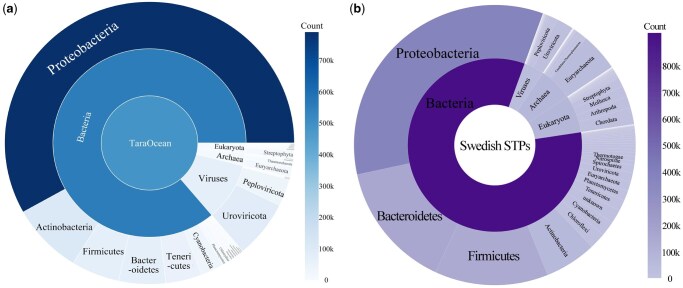
Predicted taxonomic composition by ICCTax for (a) the Tara Oceans metagenomic dataset and (b) the wastewater metagenomic dataset.

We evaluated ICCTax on two real-world datasets to assess its ability to characterize microbial community structures. One was a Tara Oceans dataset comprising 1 468 449 contigs, and the other was a wastewater metagenomic dataset comprising 1 121 463 contigs. ICCTax splits sequences longer than 1500 bp into equal chunks and generates one prediction per sequence by averaging across chunks. The predicted taxonomic distributions are illustrated in Fig. 5.

For the Tara Oceans dataset, the phylum-level classifications predicted by ICCTax are highly consistent with observations from real marine environments. For example, *Proteobacteria* account for ∼53.98% of ICCTax’s classification results, and studies show that *Proteobacteria* are one of the most abundant and widely distributed bacterial groups in marine environments, particularly in water bodies and sediments, playing a key role in processes like nitrogen fixation and sulfur cycling (Nunes-Alves 2015). Similarly, *Uroviricota* viruses (8%) are known to infect marine plankton, promoting organic matter cycling by releasing nutrients through plankton cell lysis, which influences carbon and nutrient flow in marine food webs (Zhou *et al.* 2023). *Streptophyta* are key primary producers in coastal and offshore areas, where they perform photosynthesis and support marine food webs. Although less abundant than other groups, their critical role in primary production justifies the 1.26% prediction, especially in areas with high light availability (Tragin and Vaulot 2018). For the wastewater metagenomic dataset, ICCTax identified *Proteobacteria*, *Bacteroidetes*, *Firmicutes*, and *Actinobacteria* as dominant groups, consistent with previous reports of sewage sludge microbiomes (Nascimento *et al.* 2018, de Celis *et al.* 2020, El-Khateeb *et al.* 2023). Functionally, *Proteobacteria* are known to play central roles in pollutant degradation and nutrient cycling, including organic matter decomposition, nitrification, and denitrification. These results demonstrate that ICCTax effectively predicts microbial distributions in real datasets and is a powerful tool for advancing metagenomic research.

## 4 Conclusion

Accurate taxonomic classification is crucial for conservation, evolutionary studies, and ecosystem services. However, challenges such as genomic sequence similarity, intra-species variability, and insufficient reference data hinder classification accuracy, especially for rare or unseen species. In this study, ICCTax uses HyenaDNA as the foundation model for feature extraction and incorporates a complementary view-based hierarchical metric learning architecture and hierarchical-level compactness loss to enhance intra-class compactness. The all-in-one architecture allows for shared feature extraction and cross-layer information exchange, while a learnable weight adjustment factor optimizes learning across different levels, enhancing robustness and generalization. ICCTax outperforms baseline methods across three datasets by different constructing strategies, and its performance on the Simulated Marine Metagenomic Communities, DairyDB-16S rRNA, Tara Oceans and wastewater metagenomic datasets demonstrates its effectiveness in real-world scenarios.

Despite these advantages, ICCTax currently supports classification up to the genus level. Future work will focus on extending taxonomic resolution to finer levels such as species, while also improving classification accuracy, increasing taxonomic coverage. In addition, integrating large language models with unsupervised or semi-supervised learning strategies holds promise for using unlabeled biological data and improving generalization in OOD problems. In conclusion, ICCTax provides a robust solution for taxonomic classification, overcoming key challenges and making valuable contributions to the broader field of biological research.

## Supplementary Material

vbaf257_Supplementary_Data

## Data Availability

The ID, OOD, and Complete datasets are available at https://osf.io/qg6mv/. The DairyDB dataset is available at https://github.com/marcomeola/DAIRYdb. The Tara Oceans dataset can be accessed at http://www.ebi.ac.uk/ena/about/tara-oceans-assemblies (BioProject PRJEB7988). The wastewater metagenomic dataset can be accessed at https://www.ebi.ac.uk/ena/browser/view/PRJEB22521, with the sample ERR1414211.
