# Supplementary material for: ICCTax: a hierarchical taxonomic classifier for metagenomic sequences on a large language model
Source: Bioinform Adv. 2025 Oct 15;5(1):vbaf257. doi: 10.1093/bioadv/vbaf257 (PMC12619997; doi:10.1093/bioadv/vbaf257)
Supplement: vbaf257_Supplementary_Data [file vbaf257_supplementary_data.docx]

**ICCTax: A Hierarchical Taxonomic Classifier for Metagenomic Sequences on a Large Language Model**

Yichun Gao, Jiaxing Bai, Feng Zhou, Yushuang He, Ying Wang, Xiaobing Huang

# Supplementary Section 1: Experimental Hyperparameter Settings

Experiments were conducted on three datasets—In-Distribution (ID), Out-of-Distribution (OOD), and Complete—to evaluate taxonomic classification performance. The ID and OOD datasets were designed for controlled evaluation at the superkingdom and phylum levels, while the Complete dataset supports practical deployment across various future scenarios and extends classification to the genus level. The training configurations, including batch size, number of epochs, and optimizer settings, are listed in Table S1, based on the Complete dataset and the hardware constraints of our system (*Intel® Xeon® Gold 6148 CPU @ 2.40 GHz*, *NVIDIA GeForce RTX 2080 Ti GPU*, and *251 GiB of RAM*).

**Table S1.** Training Hyperparameters

| **Dataset** | **Batch Size** | **Select/Trian Epoch (Seed=42)** | **Optimizer**  **(****Main/ AWL, LR)** | **Optimizer**  **(Center Loss, LR)** | **Center Loss weight** $\boldsymbol{\lambda}_{\boldsymbol{\tau}}$ |
| --- | --- | --- | --- | --- | --- |
| ID | 32 | 17/20 | Adam (1×10⁻⁴) | SGD (0.5) | 0.5(superkingdom)  0.1(phylum) |
| OOD | 32 | 18/20 | Adam (1×10⁻⁴) | SGD (0.5) | 0.5(superkingdom)  0.1(phylum) |
| Complete | 24 | 24/25 | Adam (1×10⁻⁴) | SGD (0.5) | 0.5(superkingdom)  0.1(phylum)  0.01(genus) |

# Supplementary Section 2: Dataset Descriptions

1. ID dataset contains 5,738 species of 2,288 genera from 30 phyla, as shown in Figure 2(f) of the manuscript. The ID training set consists of 5,738 species, 2,288 genera, and 30 phyla, while the testing set includes 4,319 species, 1,975 genera, and 30 phyla, as shown in Figure 2(c). 2,000 sequences are randomly selected from each phylum for the test set, totaling 60,000 sequences, with the remaining sequences used for training, totaling 2,328,584 sequences. Figure 2(a) illustrates the partitioning process of the ID dataset, where the genomic sequences in the test set are from species that also appear in the training set, so the test data closely matches the training data. ID, OOD, Complete Data are available at <https://osf.io/qg6mv/>, and taxonomic information was derived from the NCBI taxonomy archive (new_taxdump_2021-04-01.zip).
2. OOD dataset contains identical 5,738 species with ID dataset. But its training set consists of 5,541 species, 2,226 genera, and 30 phyla, totaling 2,245,416 sequences, while the testing set includes 197 species, 62 genera, and 30 phyla, totaling 53,400 sequences. Figure 2(b) and 2(c) of the manuscript demonstrate that there is no overlap in genus and species between the training and testing set, meaning all the genera in the testing set have never been trained in the model before. This design emulates real-world metagenomic challenges, where classifiers must handle novel organisms with distant evolutionary relationships to known clades, a task that becomes more challenging as genomic diversity increases.
3. Complete dataset includes 5,830 species, 2,330 genera, and 54 phyla, with a total of 5,399,920 sequences. Figure 2(d) and 2(e) of the manuscript show that, compared to the ID and OOD datasets, the distribution of species and their corresponding sequences in the Complete dataset is more imbalanced. Sequences from 11 phyla and 2,175 genera with fewer than 10,000 sequences were grouped into an “unknown” class at each corresponding taxonomic level, resulting in a final label set of 44 phyla and 156 genera used for classification. Then, 2,000 sequences are randomly selected from each phylum to form the testing set, totaling 88,000 sequences, with the remaining sequences are used for training, resulting in a total of 5,311,920 sequences. This dataset helps facilitate robust evaluation in a broader genomic context.
4. To evaluate classifier performance under realistic community compositions and sequencing noise, we constructed the Simulated Marine Metagenomic Communities dataset based on phylum-level abundance profiles from three ecologically distinct marine sampling sites (P1, NP3, NP5) (Duncan et al., 2022). These sites, sampled during RV Polarstern expeditions (Martin et al., 2021), represent diverse oceanic regions: P1 originates from the Arctic Ocean, whereas NP3 and NP5 are located in the tropical and subtropical North Atlantic. To ensure broad phylogenetic coverage, at least one representative genome was selected per phylum at each site. Using *Grinder* (Angly et al., 2012), we simulated 200,000 single-end 1,500 bp shotgun reads per site under three sequencing error rates (0%, 0.5%, and 5%), reflecting realistic read distributions in marine metagenomic data.
5. The DairyDB (Meola et al., 2019) dataset consists of 10,439 16S rRNA sequences derived from microbial species present in dairy products. We kept 7,828 sequences longer than 1,400 bp, which include sequences from 3,635 species belonging to 45 phyla. Data were obtained from DAIRYdb_v1.2.4 (release date: 2020-06-04).
6. We used the Tara Oceans consortium dataset (BioProject PRJEB7988) (Sunagawa et al., 2020) assembly, which includes 1,468,449 contigs. This dataset, derived from seawater samples collected during the Tara Ocean Expedition, provides valuable data for recovering high-quality metagenome-assembled genomes (MAGs) from marine environments. The genomes used include *GCA_001756945.1.fasta*, *GCA_001756965.1.fasta*, *GCA_001756985.1.fasta*, *GCA_001757005.1.fasta*, *GCA_001757045.1.fasta*, and *GCA_001757525.1.fasta*.
7. We used the wastewater metagenomic dataset from project PRJEB22521, focusing on sample ERR1414211, which contains 1,121,463 sequences with lengths ranging from 55 bp to 140,893 bp. This dataset, derived from sludge biome samples collected in Swedish sewage treatment plants (STPs), represents highly complex and diverse microbial mixtures, providing a challenging benchmark for evaluating classifier robustness in wastewater environments.

# Supplementary Section 3: Runtime Comparison of Taxonomic Classification Methods

Table S2 shows the compute benchmark comparing ICCTax with MMSeqs2, Minimap2, Kraken2, DeepMicrobes, and BERTax across different sequence lengths (500–2,500 bp), measured per 1,000 sequences, using their recommended parameter settings. Reported runtimes represent the average of five independent runs. All experiments were conducted on the same hardware platform to ensure a fair and consistent comparison.

**Table. S2** Inference benchmarks (time and memory usage per 1,000 sequences) across sequence lengths (500–2,500 bp).

| Length | MMSeqs2 | Minimap2 | Kraken2 | DeepMicrobes | BERTax | ICCTax |
| --- | --- | --- | --- | --- | --- | --- |
| (bp) | (CPU s) | (CPU s) | (CPU s) | (CPU s) | (GPU s/Mem) | (GPU s/Mem) |
| 500 | 19.42 | 35.97 | 4.20 | 25.73 | 73.22s/10.4GiB | 11.79s/4.2GiB |
| 1,000 | 19.53 | 36.14 | 4.25 | 41.91 | 75.18s/10.4GiB | 11.81s/4.2GiB |
| 1,500 | 19.67 | 36.44 | 4.38 | 57.95 | 77.61s/10.4GiB | 11.57s/6.3GiB |
| 2,000 | 20.31 | 36.73 | 4.49 | 72.42 | 340.27s/10.4GiB | 22.50s/8.2GiB |
| 2,500 | 20.51 | 39.65 | 4.58 | 89.14 | 602.72s/10.4GiB | 34.21s/8.3GiB |

# Supplementary Section 4: Evaluation metrics

Accuracy (Acc) measures the proportion of correctly classified sequences out of the total number of sequences. It is calculated as follows:

$Acc=\frac{TP+TN}{N}$, (1)

where $TP$, $TN$, and $N$ are the true positives, true negatives, and the number of sequences.

Macro average precision (AveP) can be thought of as an extension of the single-class Area Under the Precision-Recall Curve (AUPRC) to multi-class classification tasks.

$Precision=\frac{TP}{TP+FP}$, (2)

$Recall=\frac{TP}{TP+FN}$, (3)

$AveP=\sum_{n} ({Recall}_{n}-{Recall}_{n-1}){\times Precision}_{n}$, (4)

where $FP$ is the number of false positives and $FN$ represents the false negatives, ${Recall}_{n}$ and ${Precision}_{n}$ are the $Recall$ and $Precision$ at the $n$th threshold (Zhu, 2004).

# Supplementary Section 5: ICCTax performance across different taxonomic levels on the ID, OOD, and Complete datasets


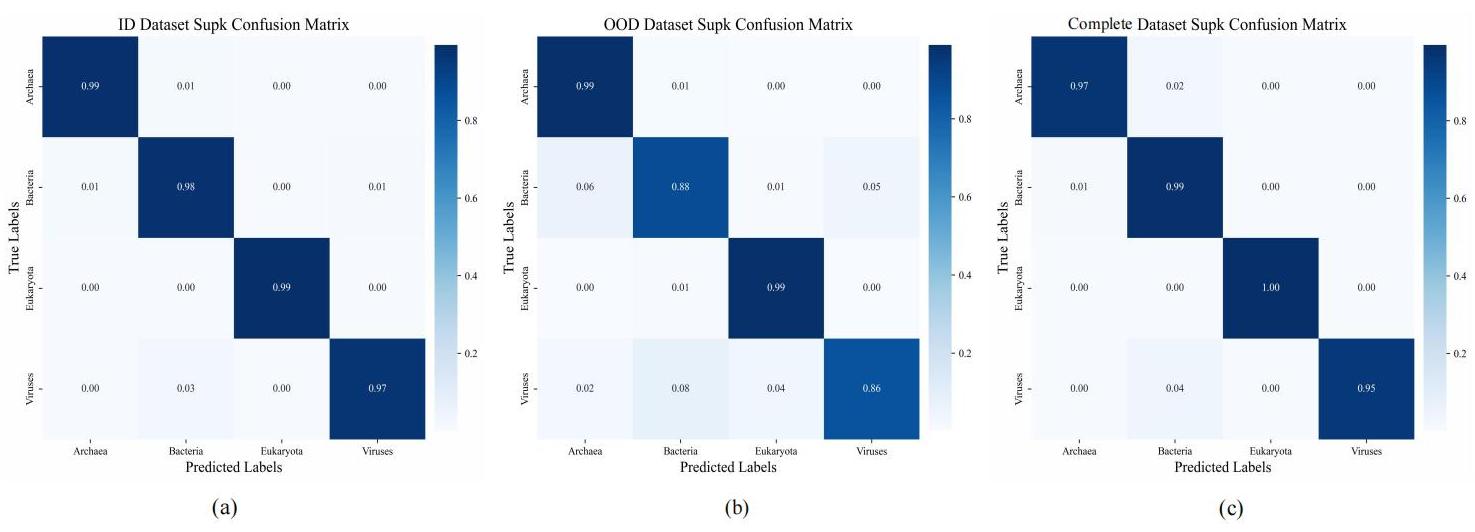

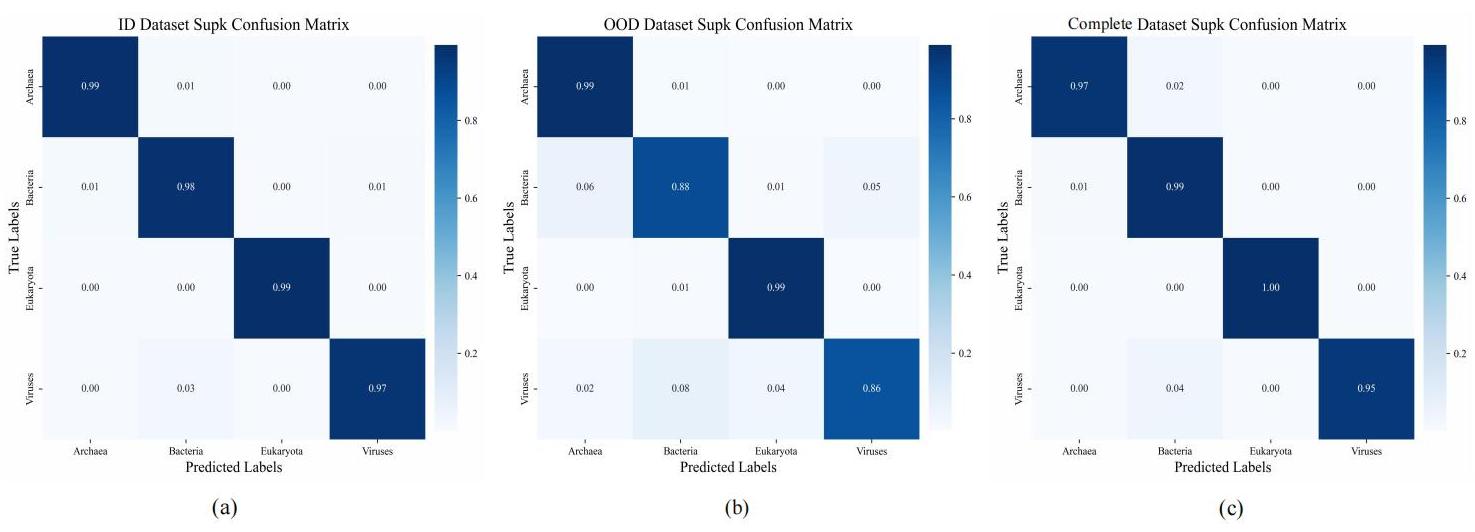


**Figure S1.** **Confusion matrices of ICCTax at the superkingdom level on the ID, OOD, and Complete datasets.** Each matrix shows the proportion of samples predicted as each class relative to the total number of samples with the corresponding true label. Values are normalized per row to reflect per-class prediction ratios.


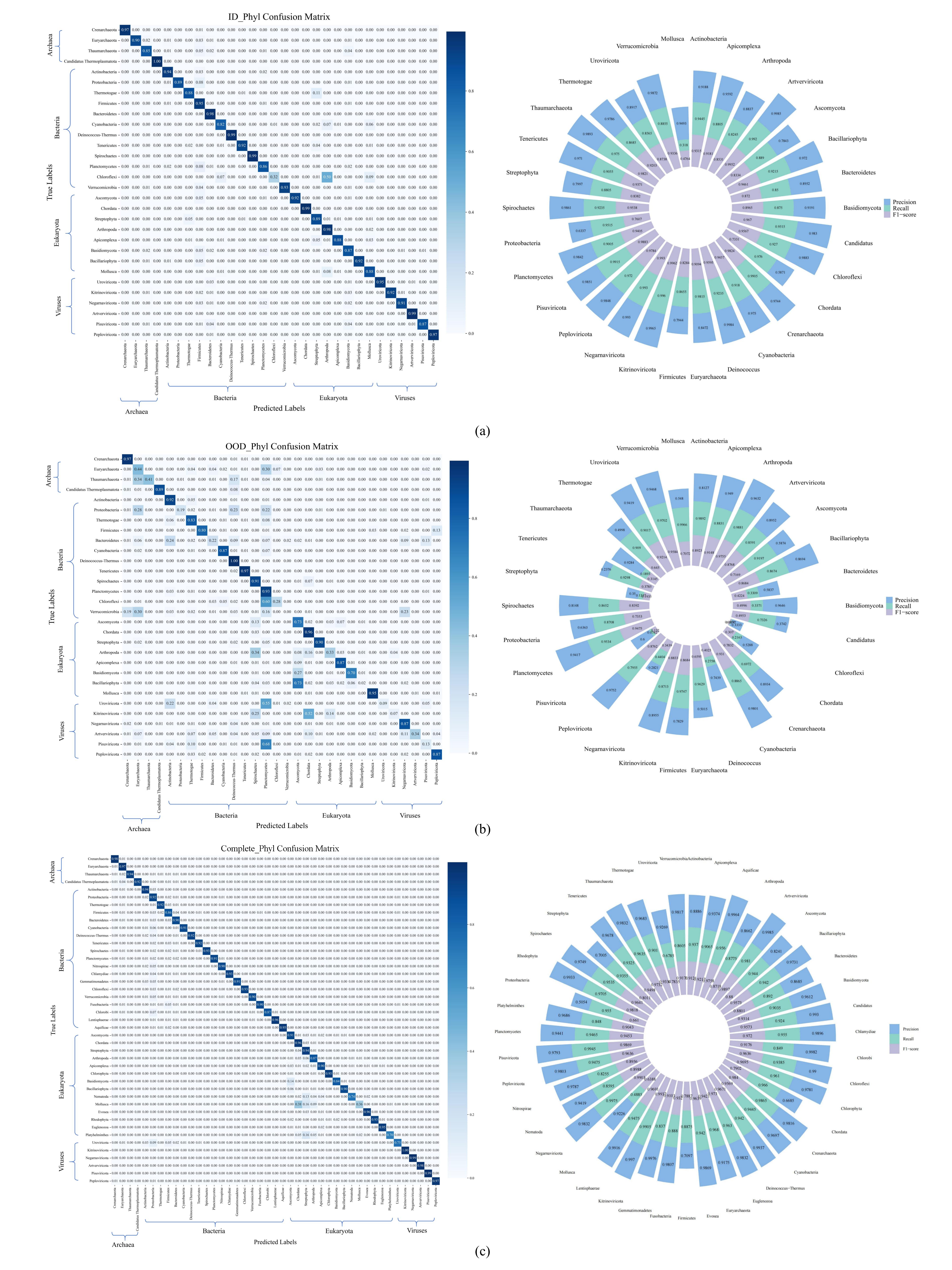


**Figure S2. Confusion matrix of ICCTax at the phylum level and the evaluation metrics (Precision, Recall, and F1-score).**

# Supplementary Section 6: AUC results for the ID, OOD, and Complete datasets

**Table. S4** Macro-averaged AUC values for ICCTax and baseline methods at the Superkingdom and Phylum levels on the ID, OOD, and Complete datasets.

|  | ID dataset | | OOD dataset | | Complete dataset | |
| --- | --- | --- | --- | --- | --- | --- |
|  | Superkingdom | Phylum | Superkingdom | Phylum | Superkingdom | Phylum |
| ICCTax | 0.9992 | 0.9985 | 0.9911 | 0.9617 | 0.9994 | 0.9988 |
| BERTax | 0.97 | 0.93 | 0.94 | 0.80 | 0.99 | 0.96 |
| DeepMicrobes | 0.98 | 0.94 | 0.81 | 0.70 | 0.98 | 0.96 |
| MMseqs2 | 0.94 | 0.93 | 0.75 | 0.71 | 0.99 | 0.96 |
| Minimap2 | 0.90 | 0.88 | 0.62 | 0.59 | 0.95 | 0.93 |
| Kraken2 | 0.90 | 0.88 | 0.62 | 0.59 | 0.95 | 0.93 |
| sourmash | 0.54 | 0.53 | 0.50 | 0.50 | 0.54 | 0.53 |

**Table. S3** Micro-averaged AUC values for ICCTax and baseline methods at the Superkingdom and Phylum levels on the ID, OOD, and Complete datasets.

|  | ID dataset | | OOD dataset | | Complete dataset | |
| --- | --- | --- | --- | --- | --- | --- |
|  | Superkingdom | Phylum | Superkingdom | Phylum | Superkingdom | Phylum |
| ICCTax | 0.9994 | 0.9985 | 0.9907 | 0.9486 | 0.9994 | 0.9988 |
| BERTax | 0.97 | 0.93 | 0.94 | 0.80 | 0.99 | 0.98 |
| DeepMicrobes | 0.98 | 0.94 | 0.81 | 0.70 | 0.98 | 0.96 |
| MMseqs2 | 0.94 | 0.93 | 0.75 | 0.71 | 0.99 | 0.96 |
| Minimap2 | 0.90 | 0.88 | 0.62 | 0.59 | 0.95 | 0.93 |
| Kraken2 | 0.90 | 0.88 | 0.62 | 0.59 | 0.95 | 0.93 |
| sourmash | 0.54 | 0.53 | 0.50 | 0.50 | 0.54 | 0.53 |

# Supplementary Section 7: ICCTax performance CIs (bootstrap) on Acc and AveP are in the ID and OOD datasets

The 95% bootstrap confidence intervals (CIs) for Accuracy (Acc) and macro average precision (AveP) are reported in Figs. S3–S4. The CIs are narrow (e.g., Supk Accuracy in OOD: [0.918, 0.923]; Phyl Accuracy: [0.612, 0.619]), confirming the statistical robustness of the reported metrics. Consistent results across both Acc and AveP further indicate that ICCTax maintains stable performance under resampling variability. Reliability diagrams (right panels of Figs. S3–S4) additionally show that ICCTax predictions are well calibrated in the ID dataset (ECE = 0.005 at the phylum level), whereas calibration is weaker in the OOD dataset (ECE = 0.212), which is expected given the higher difficulty of classifying unseen taxa. These results collectively reinforce the robustness and credibility of the evaluation.


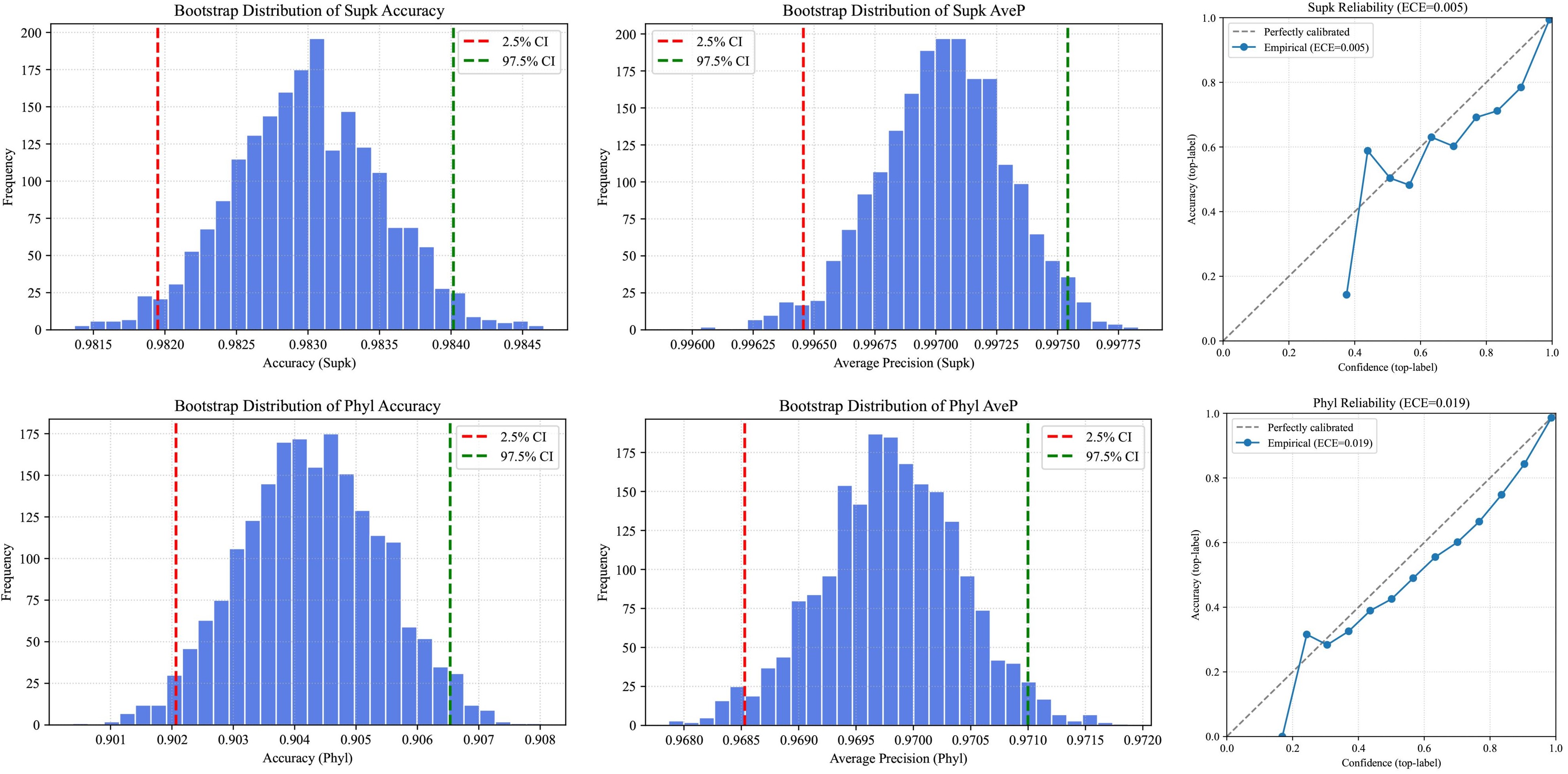


**Figure S3. 95% of ICCTax's CIs (bootstrap) on Acc and AveP are in the ID dataset**


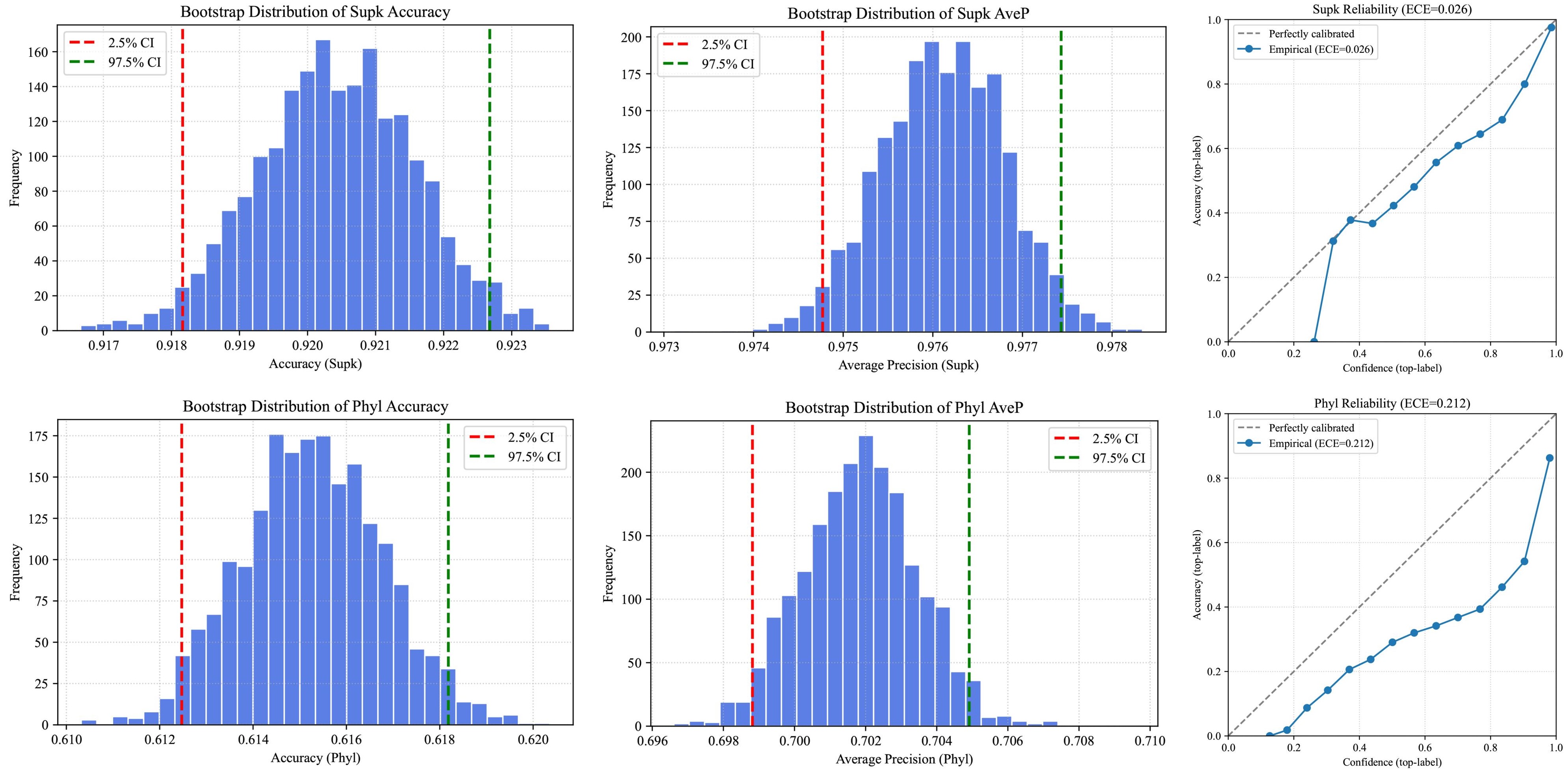


**Figure S4. 95% of ICCTax's CIs (bootstrap) on Acc and AveP are in the OOD dataset.**

# Supplementary Section 7: ICCTax performance on the Simulated Marine Metagenomic Communities dataset


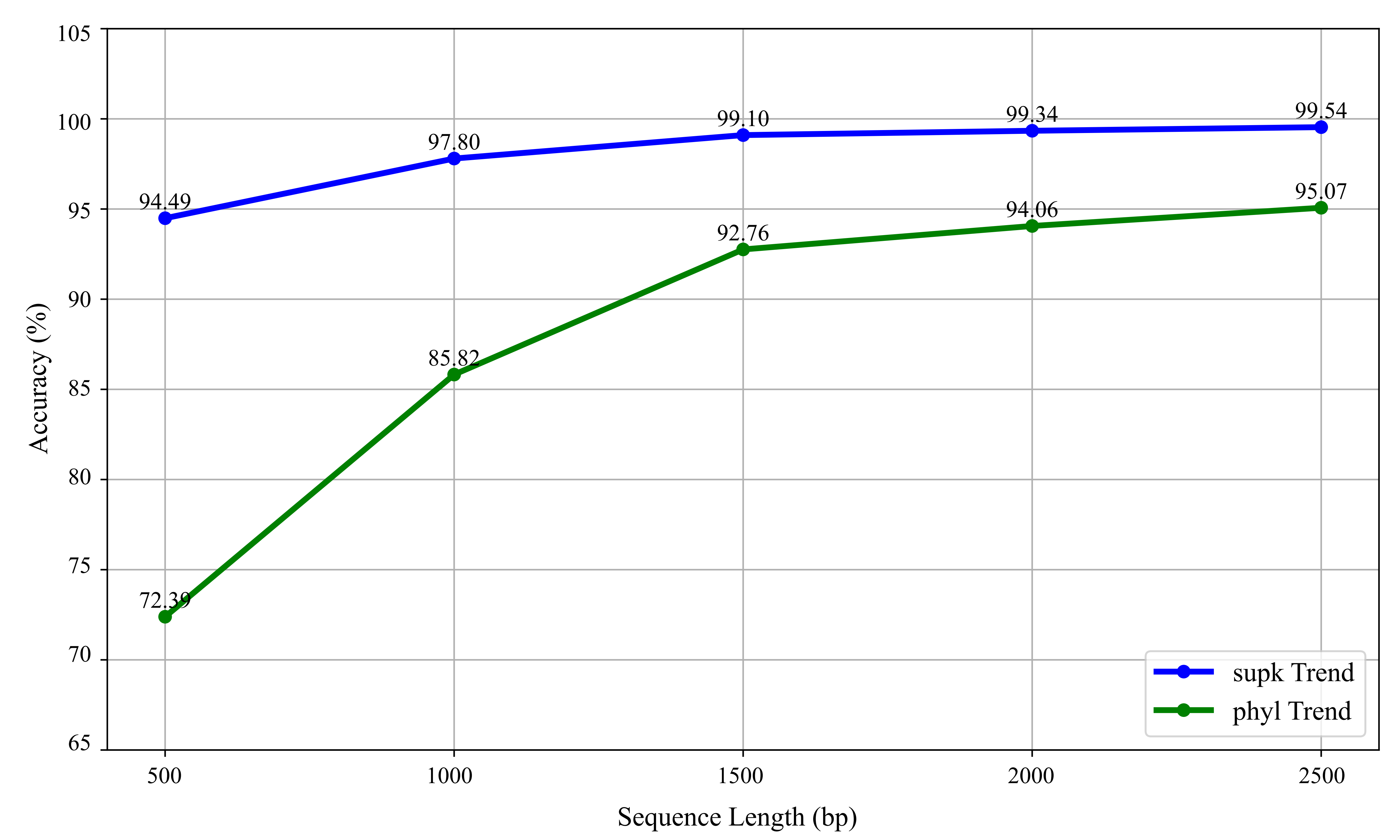


**Figure S6. ICCTax accuracy at superkingdom and phylum levels across sequence lengths (500–2,500 bp) on Grinder-simulated data (P1, 5% error).**


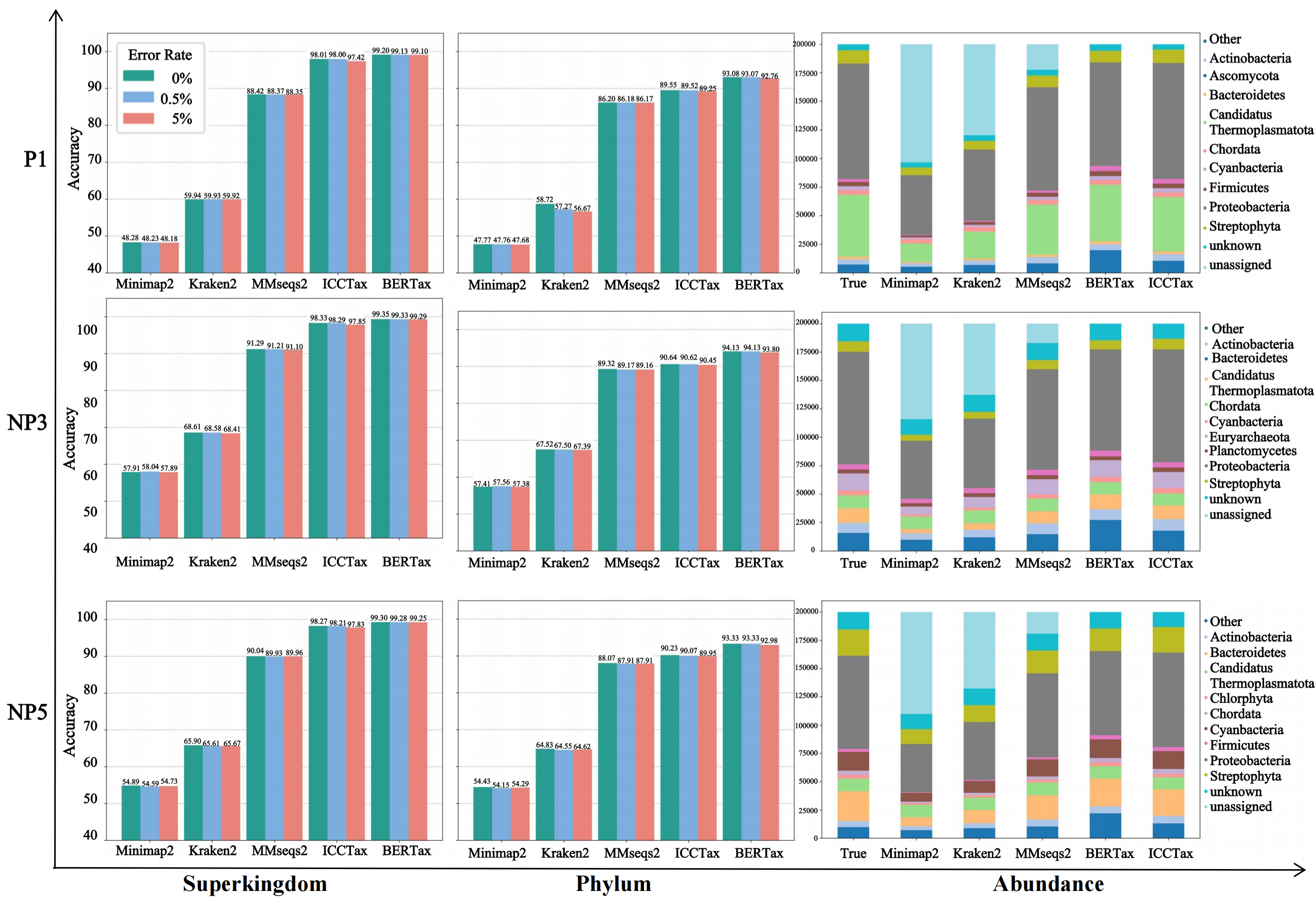


**Figure S5. Classification accuracy and phylum-level abundance comparisons on the Simulated Marine Metagenomic Communities datasets.** Each row corresponds to one of the three representative sampling sites: P1 (Arctic Ocean), NP3, and NP5 (tropical/subtropical North Atlantic). Columns represent classification accuracy at the superkingdom and phylum level under three sequencing error rates (0%, 0.5%, and 5%), and predicted phylum-level abundance distributions (right) under the 0% error rate. True abundance profiles are shown alongside the predicted results to assess compositional accuracy across methods.

# Supplementary Section 8: Ablation Study


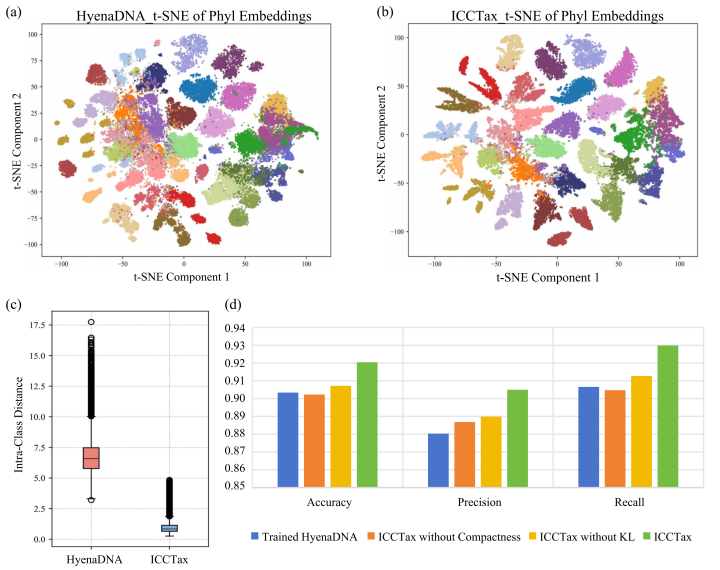


**Figure S7.** (a) and (b) t-SNE visualizations of DNA sequence data at the phylum level, generated by the trained HyenaDNA and ICCTax models, respectively. (c) Boxplots showing intra-class distances from the mean center, where the mean center is calculated by averaging all samples within each class in the embedding space. (d) Ablation study of the ICCTax components on OOD testing datasets.

To further examine the impact of different distance measures in our complementary-view-based metric learning, we conducted additional experiments on the OOD dataset using Kullback–Leibler Divergence (KLD) (Kullback, 1997), Jensen–Shannon Divergence (JSD)(Lin, 2002), Hellinger Distance (HD)(Hellinger, 1909), and Earth Mover’s Distance (EMD)(Rubner et al., 2000). All models were trained and evaluated under identical settings to ensure fairness.

As shown in Table S3, KLD achieved the best overall performance, particularly at the phylum level (Acc = 61.53, AveP = 70.17). JSD and HD yielded slightly lower but comparable results, whereas EMD performed substantially worse (Acc = 48.86, AveP = 60.47). These findings justify our choice of KLD as the primary divergence measure, while also suggesting that JSD and HD could serve as reasonable alternatives.

KLD’s superior performance can be explained by two factors. First, its asymmetry introduces directional sensitivity, making it well suited for aligning predictions of original and reverse-complement sequences. Second, KLD places greater emphasis on discrepancies in low-probability regions, which is advantageous for capturing rare or underrepresented taxa in imbalanced and OOD scenarios. By contrast, JSD and HD are symmetric and smoother divergences that may blur subtle differences, while EMD assumes an underlying geometric structure that may not align with the categorical probability space of taxonomic predictions.

**Table S5. Comparison of divergence and distance metrics on the OOD dataset**

| OOD dataset | | | | |
| --- | --- | --- | --- | --- |
|  | Superkingdom | | Phylum | |
|  | Acc | AveP | Acc | AveP |
| KLD | **92.04** | **97.61** | **61.53** | 70.17 |
| JSD | 91.03 | 96.84 | 61.02 | **70.39** |
| HD | 91.52 | 96.05 | 60.46 | 69.20 |
| EMD | 89.36 | 95.77 | 48.86 | 60.47 |

# Supplementary Section 9: Performance on DairyDB-16S rRNA


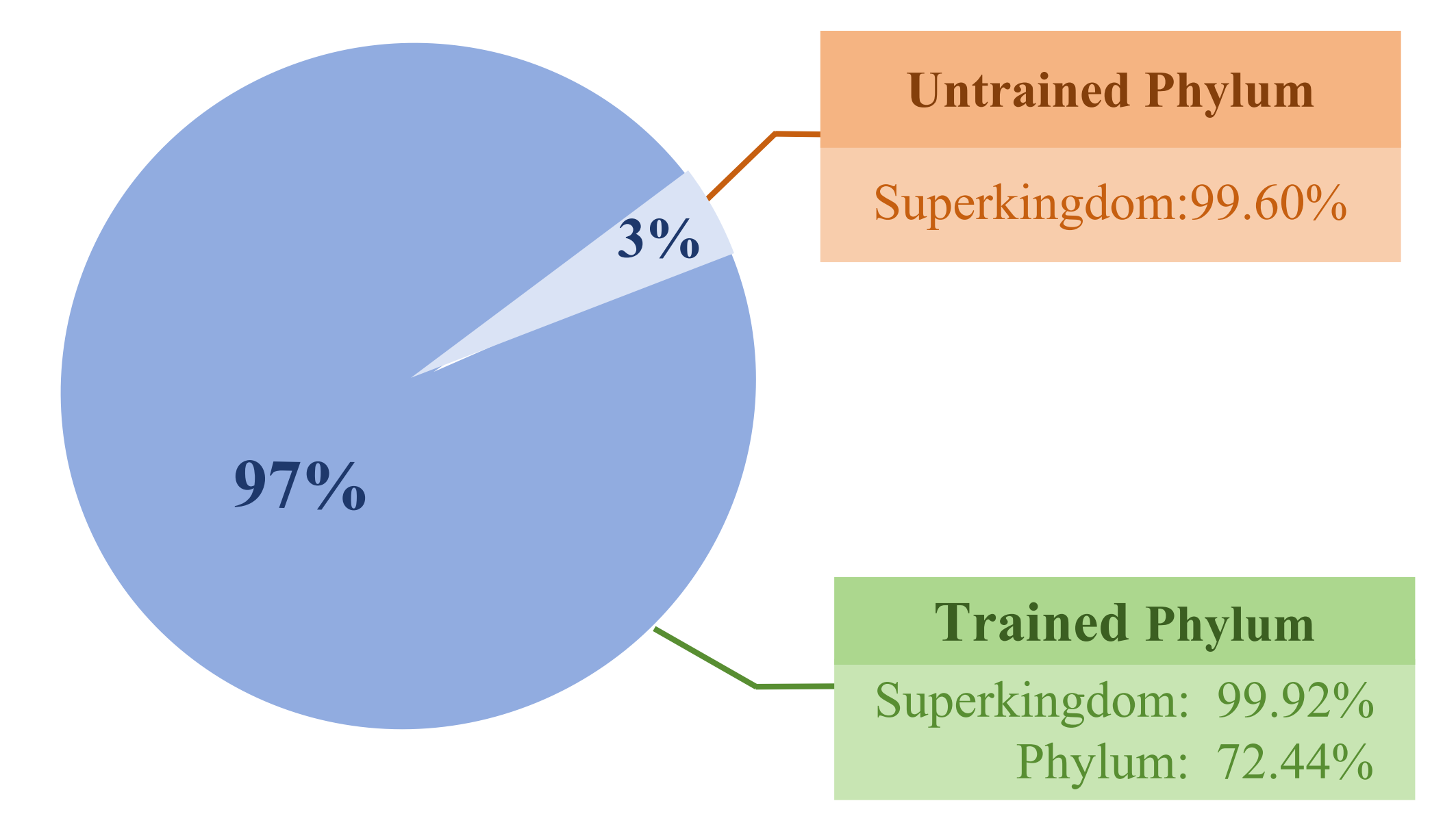


**Figure S8.** (a) and (b) t-SNE visualizations of DNA sequence data at the phylum level, generated by the trained HyenaDNA and ICCTax models, respectively. (c) Boxplots showing intra-class distances from the mean center, where the mean center is calculated by averaging all samples within each class in the embedding space. (d) Ablation study of the ICCTax components on OOD testing datasets.

# Supplementary Section 10: Interpretability Analysis of ICCTax

Interpretability is a recognized challenge for deep learning–based classifiers. Unlike BERTax, which provides attention-based transparency, ICCTax employs the HyenaDNA operator, where long-range dependencies are captured by implicit convolutional filters rather than explicit token–token attention. Consequently, conventional attention visualizations are not directly applicable, and alternative interpretability strategies are required.

To address this, we applied In-silico Mutagenesis (ISM) to quantify the position-wise contributions of nucleotides. Six representative 1,500 bp sequences were randomly selected from different genera within the same phylum, and ISM-4 was used to compute Δlogit scores across sequence positions. For each sequence, the top-*K* subsequences (25 bp windows) exhibiting the largest deviations from the cohort mean (gray baseline) were identified as high-impact regions. These subsequences were subsequently aligned against the NCBI BLAST database (Johnson et al., 2008).


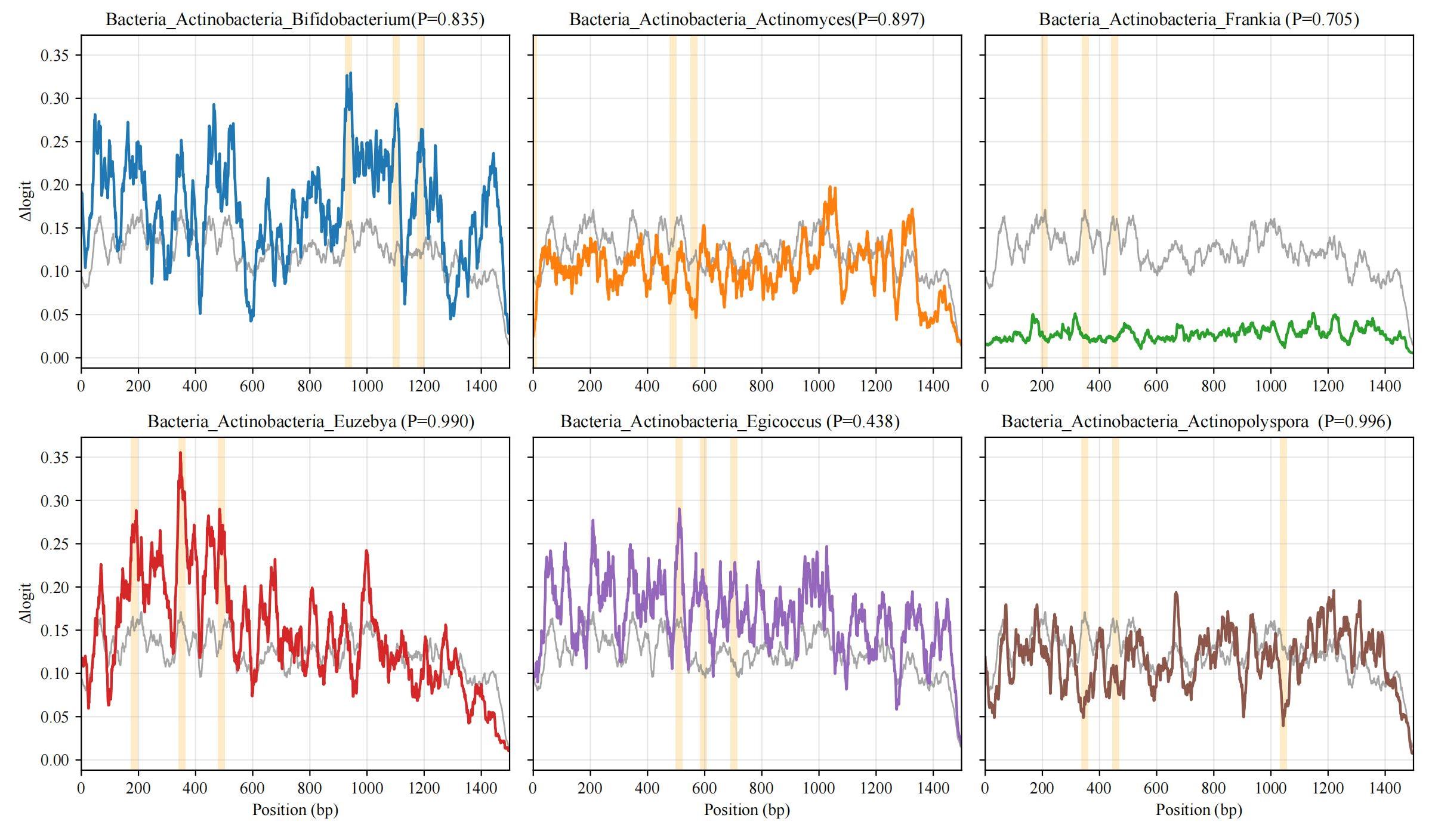


**Figure S9.** In-silico Mutagenesis (ISM) analysis of sequences sampled from different genera within the phylum *Actinobacteria*. The y-axis shows Δlogit values reflecting the change in model confidence when mutations are introduced, and the x-axis represents the sequence position (bp). The gray curve indicates the cohort mean across six sampled sequences, while the colored curves represent individual sequences from different genera.

As shown in Figure S9, ISM highlights distinct sequence windows that strongly influence model predictions. To validate the biological relevance of these high-impact regions, we further examined the genus Bifidobacterium. The BLAST results for its top-3 subsequences (Table S4) show consistent alignment to Bifidobacterium or phylogenetically related genera with high scores and coverage. This demonstrates that ICCTax predictions are not driven by random noise but rather by biologically meaningful motifs. Compared with conventional attention maps, the ISM-based framework provides both positional importance and direct biological validation, thereby strengthening the interpretability of ICCTax.

**Table. S6** Representative BLAST results of top-3 high-impact subsequences identified by ISM in *Bifidobacterium* (phylum *Actinobacteria*).

| Rank | Start_bp | End_bp | Subseq | Taxonomy  Genus | Max  Score | Query  Cover | E_value |
| --- | --- | --- | --- | --- | --- | --- | --- |
| 1 | 923 | 948 | ACGCGGCCCGAA-  TCAGGTTCCTGCG | Bifidobacterium | 50.1 | 100% | 0.003 |
|  |  |  |  | Streptomyces | 38.2 | 76% | 12 |
|  |  |  |  | Saccharopolyspora | 36.2 | 72% | 47 |
|  |  |  |  | Nocardia | 36.2 | 72% | 47 |
| 2 | 1089 | 1114 | CAATATCGTAAA-  TCGGCATAAT CCC | Bifidobacterium | 50.1 | 100% | 0.003 |
|  |  |  |  | Brevibacillus | 38.2 | 76% | 12 |
| 3 | 1175 | 1200 | ATCCATCTCCAA-  AATCGTTGATTTT | Bifidobacterium | 50.1 | 100% | 0.003 |
|  |  |  |  | Enterococcus | 38.2 | 76% | 12 |
|  |  |  |  | Lactobacillus | 38.2 | 76% | 12 |

# References

ANGLY, F. E., *et al.* (2012). Grinder: a versatile amplicon and shotgun sequence simulator. *Nucleic acids research,* 40**,** e94-e94.

DUNCAN, A., *et al.* (2022). Metagenome-assembled genomes of phytoplankton microbiomes from the Arctic and Atlantic Oceans. *Microbiome,* 10**,** 67.

HELLINGER, E. (1909). Neue begründung der theorie quadratischer formen von unendlichvielen veränderlichen. *Journal für die reine und angewandte Mathematik,* 1909**,** 210-271.

JOHNSON, M., *et al.* (2008). NCBI BLAST: a better web interface. *Nucleic acids research,* 36**,** W5-W9.

KULLBACK, S. (1997). *Information theory and statistics*, Courier Corporation.

LIN, J. (2002). Divergence measures based on the Shannon entropy. *IEEE Transactions on Information theory,* 37**,** 145-151.

MARTIN, K., *et al.* (2021). The biogeographic differentiation of algal microbiomes in the upper ocean from pole to pole. *Nature Communications,* 12**,** 5483.

MEOLA, M., *et al.* (2019). DAIRYdb: a manually curated reference database for improved taxonomy annotation of 16S rRNA gene sequences from dairy products. *BMC genomics,* 20**,** 1-16.

RUBNER, Y., *et al.* (2000). The earth mover's distance as a metric for image retrieval. *International journal of computer vision,* 40**,** 99-121.

SUNAGAWA, S., *et al.* (2020). Tara Oceans: towards global ocean ecosystems biology. *Nature Reviews Microbiology,* 18**,** 428-445.

ZHU, M. (2004). Recall, precision and average precision. *Department of Statistics and Actuarial Science, University of Waterloo, Waterloo,* 2**,** 6.
